# How can evidence-based interventions give the best value for users in social services? Balance between adherence and adaptations: a study protocol

**DOI:** 10.1186/s43058-020-00005-9

**Published:** 2020-02-25

**Authors:** Henna Hasson, Hedvig Gröndal, Åsa Hedberg Rundgren, Gunilla Avby, Håkan Uvhagen, Ulrica von Thiele Schwarz

**Affiliations:** 1 Procome Research Group, Department of Learning, Informatics, Management and Ethics, Medical Management Centre, Karolinska Institutet, SE-171 77 Stockholm, Sweden; 2 Unit for Implementation and Evaluation, Center for Epidemiology and Community Medicine (CES), Stockholm County Council, SE-171 29 Stockholm, Sweden; 3 Stockholm Gerontology Research Center, Stiftelsen Stockholms läns Äldrecentrum, Sveavägen 155, 113 46 Stockholm, Sweden; 4 FoU Nordväst, Research and Development Center for social services in northwestern Stockholm County Council, Oppegårdsstråket 12, SE-191 86 Sollentuna, Sweden; 5 Jönköping Academy for Improvement of Health and Welfare, School of Health and Welfare, Jönköping University, Jönköping, Sweden; 6 Stockholm Research and Development Unit for Elderly Persons (FoU nu), Stockholm County Council, 177 31 Järfälla, Sweden; 7 School of Health, Care and Social Welfare, Mälardalen University, Box 883, 721 23 Västerås, Sweden

**Keywords:** Social services, Social work, Adaption, Adherence, Adaption-adherence dilemma, Evidence-based interventions, Evidence-based practice, Decision support

## Abstract

**Background:**

Using evidence-based interventions (EBIs) is a basic premise of contemporary social services (e.g., child and family social services). However, EBIs seldom fit seamlessly into a specific setting but often need to be adapted. Although some adaptions might be necessary, they can cause interventions to be less effective or even unsafe. The challenge of balancing adherence and adaptations when using EBIs is often referred to as the adherence and adaptation dilemma. Although the current literature identifies professionals’ management of this dilemma as problematic, it offers little practical guidance for professionals. This research aims to investigate how the adherence and adaptation dilemma is handled in social services and to explore how structured decision support can impact the management of the dilemma.

**Methods:**

The design is a prospective, longitudinal intervention with a focus on the feasibility and usefulness of the structured decision support. The project is a collaboration between academic researchers, embedded researchers at three research and development units, and social service organizations. A multi-method data collection will be employed. Initially, a scoping review will be performed, and the results will be used in the development of a structured decision support. The decision support will be further developed and tested during a series of workshops with social service professionals. Different forms of data—focus group interviews, questionnaires, and documentation—will be used on several occasions to evaluate the impact of the structured decision support. Qualitative and quantitative analysis will be performed and usefulness for practice prioritized throughout the study.

**Discussion:**

The study will contribute with knowledge on how the adherence and adaption dilemma is handled and experienced by social service professionals. Most importantly, the study will generate rich empirical data on how a structured decision support impacts professionals’ management of adherence and adaptions. The goal is to produce more strategic and context-sensitive implementation of EBIs in social service, which will increase value for service users.

Contribution to the literature

- The study contributes with knowledge on how the balance between adherence and and adaptions of EBIs is achieved by social service professionals.
- The study contributes with knowledge about whether the adherence and adaptation dilemma relates to professionals’ experience of cognitive and emotional demands at work.
- A structured decision support for management of the adherence and adaption dilemma will be developed and tested.

## Background

Provision of good-quality social services, which are equally distributed to all citizens in need, is a common goal for decision-makers and service providers [1]. Using evidence-based interventions (EBIs) is considered one way to achieve this [2]. Although there is an ongoing discussion within social service research and practice concerning what kind of knowledge should count as evidence and how evidence should reach social workers [1, 3], there is a clear expectation that social service practice should be based on evidence [4, 5]. In Sweden, this is mirrored in a national policy of basing social services (i.e., social support to children and families, individuals with disabilities, people with substance abuse problems, and older people) on evidence, together with professionals’ experience and clients’ preferences [6].

However, using EBIs in social services has shown to be challenging. Two interlinked problems have occurred. First, professionals in social services are struggling with determining how to use their own specific expertise as well as clients’ experiences and preferences in relation to evidence and EBIs [7–9]. Although scientific models suggest how this can be done in theory (e.g., [10]), the practice lacks clear and hands-on guidance on how different knowledge sources are best combined when making decisions [7, 11, 12]. Second, EBIs seldom fit seamlessly into a specific setting [13]. The context in which an EBI was developed and tested often differs in substantial ways from the context in which it is applied. For instance, workforce, provider capacity, staff training, welfare systems, resources, and service user characteristics can be different [14]. These misfits between contexts mean that EBIs often need to be adapted to fit the new context [15, 16]. Most EBIs used in social services will be adapted, to various degrees [17]. Studies from other areas show that adaptation is the rule rather than the exception [18]. An accumulation of findings has shown that between 44 and 88% of users adapt the procedure, dosage, content, format, and/or target group when using EBIs (e.g., [19–22]).

Although adaptions of EBIs can have beneficial effects for clients, and even might be necessary to implement an EBI, adaptations are not unproblematic. Adaptations can cause an EBI to be less effective or even unsafe [23]. In fact, high adherence has been related to better client outcomes in a number of studies (e.g., 20). Adaptations can also induce unwanted variation in the services provided to clients, as compared to the original EBI and between different service users. Thus, adaptations might increase the risk of inequalities in service provision [24]*.* The challenge of balancing adherence and adaptations when using EBIs is often called the adherence and adaptation dilemma [25]. At heart, this dilemma concerns how evidence can be applied in a specific setting. More specifically, it is concerned with the extent to which an EBI needs to adhere to the original version of the EBI and the extent to which purposeful changes to the EBI in response to restraints and possibilities in the local setting are acceptable or even desirable.

### Literature on adherence and adaptations

Most of the literature on the adherence and adaptation dilemma is conceptual or theoretical, building convincing arguments for either the importance of high adherence or the burning need for local adaptations [23, 26]. As described above, some empirical studies also show that adaptations are common in practice (e.g., [13]). Fewer studies show why and how adaptations are done in practice, although in general, issues of adherence and adaptation tend to be handled in an ad hoc way [19, 27].

In the literature, we identified five main ways of managing the adherence and adaptation dilemma that can be problematic for the outcomes of an EBI. First, adaptations often seem to be conducted reactively as an impulsive response to constraints in the context (e.g., time pressure). This is problematic, because research shows that proactive adaptations based on a thoughtful analysis of the EBI, the context in which it was developed and tested, and the context in which it will be implemented tend to give better client outcomes [19, 22]. Second, decisions about adaptations are often made in response to practical restraints (that is, to get the EBI in place) rather than to provide a better fit with the target group [13]. Third, decisions are often made by individuals rather than based on discussions in work teams [28]. This increases the risk of unwanted variation and, in the end, inequalities in the provided services. Fourth, decisions regarding adherence and adaptations are often made without careful consideration of how the adaptation will affect the outcomes for clients [22]. Finally, adaptations are often made without consideration of how they will affect the EBI’s core components—that is, without ensuring that the active ingredients or core elements that make the EBI effective remain [28].

Although the literature identifies a number of problems related to how the adherence and adaption dilemma is approached in practice, it offers little practical guidance for professionals’ management of it. A call has been made for practical tools for managing the adherence and adaptation dilemma [29], emphasizing the need for solutions in the area. The current study is a response to that call. The study makes use of Lee et al.’s [29] Planned Adaptation Model and Hasson and von Thiele Schwarz’s Useful Evidence Model [30]. The Planned Adaptation Model was developed to support public health professionals in identifying core components in an EBI and determining differences between their own setting and the setting in which the EBI was originally developed. Although this model can provide some guidance for professionals, it might not be directly applicable in a social service setting. First, it is directed toward large public health programs, focusing on understanding population characteristics rather than main concerns in social service settings, such as differences among organizations (e.g., in resources, size, and staff education levels). Second, and most importantly, the model does not give guidance on how to conduct proactive, goal-oriented adaptations instead of reactive responses to practical restraints. Hence, the model is probably insufficient to solve the five problems in managing the adherence and adaptation dilemma, discussed above. The Useful Evidence Model [30] adds to Lee et al.’s model by acknowledging a broader set of contextual factors and by giving hands-on guiding principles for collaboratively managing adherence and adaptations in a work team. This model was developed in cooperation with practitioners from mental health care and pediatric care but remains to be tested in, and potentially modified to fit, social service settings [30].

### Cognitive and emotional demands among professionals

Lack of practical support for social service professionals’ management of the adherence and adaptation dilemma [30] means that they are left on their own with the dilemma, which can act as both a cognitive and emotional stressor for professionals [31]. The literature is scarce regarding cognitive and emotional demands among professionals when dealing with the adherence and adaptation dilemma. It has been highlighted that this dilemma causes significant tension for professional groups with a deep respect for scientific principles [25], but we have found no empirical studies that directly address this. A related finding, however, is that rule-practice gaps, such as when professionals are unable to act according to legal requirements or feel forced to either break rules to deliver high-quality service or act according to guidelines that are misaligned with their professional values, are a source of ethical distress for staff [31, 32]. In terms of cognitive demands, clinical guidelines have the potential to both off-load and increase cognitive demands, depending on how well the level of detail matches professionals’ needs [33]. Both too much and too little guidance tend to be problematic, which, in reference to the adherence and adaptation dilemma, might reflect the balance between adherence to specific protocols versus leaving too much room for adaptations without sufficient guidance. In addition, practitioners who receive support to adopt an EBI (possibly including support to manage the adherence and adaptation dilemma) have a better well-being than those who are not supported [34]. Thus, there is a need for studies investigating the relationship between the adherence and adaptation dilemma and emotional and cognitive demands among staff.

In sum, the adherence and adaption dilemma is an inevitable part of the implementation of EBIs in social services, and no practical support is currently available to help social service professionals manage this dilemma. Thus, there is a need to explore this dilemma in social service settings, especially regarding how to support professionals in managing the dilemma in daily professional practice.

### Aim and research questions

The aim of this research is to investigate how the adherence and adaptation dilemma is handled in social services and to explore how a structured decision support to social service professionals impacts how the dilemma is managed.

The following research questions (RQs) will be addressed:

1. How is the adherence and adaptation dilemma managed?
  - How is the dilemma related to social service professionals’ experiences of cognitive and emotional demands?
2. How does structured decision support impact how the adherence and adaptation dilemma is managed? The focus is on:
  - The EBI: Does it support professionals in identifying core components?
  - The context: Does it support professionals in identifying differences between their context and the context in which the EBI has been used?
  - The adaptations: Which adaptations are made, and to what extent do these align with the EBI’s goals?
  - The knowledge sources: How are professionals’ expertise and consideration of clients’ needs expressed in the process?
  - The support: Does the support decrease social service professionals’ experience of cognitive and emotional demands?
3. How does participation in research-practice collaboration affect social service professionals’ attitudes toward research and scientific knowledge? Do they experience more possibilities to impact social services research?

### Theoretical approach

The project takes its starting point from the notion of professionals’ discretion and how it is exercised when using general knowledge (evidence) in a specific work situation and context. Social service professionals have varying degrees of autonomy and power to decide how to carry out tasks in daily practice. To understand the tension that professionals in the welfare sector often deal with, we make use of Lipsky’s theory of street-level bureaucracy [35]. On the one hand, welfare organizations provide highly scripted regulations and goals to the professionals. However, on the other hand, professionals also need to improvise and be responsive to individual cases. They need to navigate these tensions given the boundaries of their professional values and the available organizational resources. According to Lipsky’s theory, professionals in welfare organizations often lack the necessary resources (e.g., time, information) to provide the highest quality services to each client. Professionals manage this by creating routines and psychologically simplifying both clients’ problems and their environment. Thus, this theory will offer valuable insights into the discretion of social service professionals and how this is exercised when dealing with the adherence and adaptation dilemma.

## Methods

### Participatory approach

One of the study’s basic premises is that it should meet the needs and resources of the social service organizations involved, produce relevant and actionable findings, and be of good quality to add to the scientific literature [36]. Thus, the proposed design will be discussed and further developed in interaction with the local stakeholders as the project unfolds. In doing so, the project builds on a participatory research approach (e.g., [37]).

In contrast to a traditional approach, in which researchers first produce research evidence and then disseminate it and implement it into practice, we use a constructive approach to knowledge development [37]. With this approach, knowledge is assumed to be developed through interactions between various stakeholders, resulting in knowledge that is more relevant in practice. It is also believed to facilitate dissemination and implementation of the findings because the barriers to implementation have been reduced in the early stages of knowledge development, rather than being managed upstream [36].

More specifically, the project is a collaboration between academic researchers (HH, UvTS, HG), embedded researchers at three research and development (R&D) units (ÅHR, GA, HU), and social service organizations. In Sweden, social services are the responsibility of the local municipalities. The social services include social support to children and families, support for individuals with disabilities or substance abuse problems, and eldercare (including nursing homes and home help services). The R&D units operate in specific geographical areas and have established relationships with the local social service organizations. Thus, they have knowledge about the service providers’ needs and resources.

### Study design

This is a prospective, longitudinal intervention study that will be conducted in regular social service practices rather than a controlled research context [38]. The focus is on understanding how the adherence and adaptation dilemma is handled under ordinary conditions and on exploring whether and how this can be improved with a structured decision support. Although this is an intervention study, the objective is not to conduct an effect evaluation. Instead, we follow recent calls in evaluation science to start evaluations based on the needs of the practice, focusing on usefulness first and conducting effect evaluations with a stricter set of requirements later (e.g., [38]). Thus, in addition to answering the research questions, this project might also produce an intervention that can be further tested in future research.

#### Literature review

A literature review on the adherence and adaptation dilemma in social services will be conducted as a first project activity. The findings of the review will be incorporated into the decision support. A scoping review methodology will be chosen, given the immaturity of the field [39]. This methodology is valuable for exploring the kinds of research that has been done in a field, and unlike systematic reviews, the question is often wider, and studies with all types of designs are included. The review will follow the steps outlined by Armstrong et al. [39], and the analysis will be descriptive (qualitative) rather than statistical. Relevant publications will be identified through searches in electronic databases, through searches of reference lists and of key journals. Inclusion criteria will be developed during the process and not determined beforehand. Information from included studies will be extracted and organized thematically. Themes will be inductively developed from the empirical data during the analytical process.

#### The intervention

The intervention is a structured decision support for social service professionals for managing adherence and adaptations in their daily practice. The structured decision support is based on the Planned Adaptation Model [29] and the Useful Evidence Model [30]. In addition, the results of the literature review will be incorporated into the decision support.

Based on these models, the structured decision support builds on four guiding principles for managing adherence and adaptations: (1) adaptations of any EBI should be carefully planned, with the value for service users as a goal (rather than, e.g., organizational constraints); (2) adherence and adaptation are not opposites but can co-occur (i.e., by explicating how flexibly the EBI’s core components can be applied); (3) the amount and type of research on the effectiveness of the EBI impact how it should be managed (e.g., clear evidence showing better outcomes when the EBI is used with high adherence indicates that adherence is motivated); and (4) adaptations to the local context might also be needed for an EBI to have a chance to function and produce positive outcomes for the service users.

As a first step, manuals and worksheets will be developed to form the structured decision support. These materials will be pilot tested with social service professionals and modified if needed. After the piloting, a number of workshops will be conducted to guide the professionals in the use of the structured decision support (see below). An electronic decision support system might also be developed and tested if interest and need among the social service organizations exist.

#### Participants

The 3 R&D units operate in different geographical areas involving a total of approximately 500 social service organizations in the Stockholm area. These social service organizations are the population from which the participants will be recruited. The R&D units already have established channels to these organizations.

All of the social service organizations will receive information about the project from the R&D units, and organizations that are about to implement an EBI will continuously be invited to use the structured decision support in a series of workshops. These workshops constitute the backbone of the study and are where the data will be collected.

EBIs will be defined as methods or programs that have been shown to be effective in scientifically rigorous evaluations. Nevertheless, we will not exclude any organization based on the level of evidence for the interventions, as the level of evidence for existing interventions varies considerably between different areas of social services, and we aim to contribute to practice such as it is. Moreover, the aim is that participation will contribute to building capacity for managing adherence and adaptation beyond the specific intervention at hand, thus making the knowledge recyclable. Furthermore, we will not exclude any type of social services. Thus, the study covers all areas of social services in which EBIs are implemented (e.g., support for children, families, individuals with disabilities or substance abuse problems, and older people, including nursing homes and home help services). This means that we will include multiple social service organizations, including different types of services, geographical areas that vary in size, services provided, and characteristics of the social service users. This design will increase the chances that the results will be transferable to other organizations, thus increasing the generalizability of the findings.

The participating social service organizations will be asked to identify individuals who are likely to be involved in the management and use of the particular EBI (e.g., managers, professionals, individuals responsible for a certain EBI) for participation in the workshop. This should include those who will be working with the EBI as well as people who know the context in which it will be applied and those with the mandate to change the contextual factors, if needed.

Based on the total amount of social service organizations in the area and our previous experiences with conducting similar methodological workshop series, we estimate that each R&D unit will conduct 2 to 3 series of workshops each, resulting in a total of 6–9 workshop series during the project (excl. pilot cases). In each of these, we anticipate that 5–7 work teams will participate, with approximately 3–5 individuals on each team. This would result in 45–63 work teams and 90–315 individuals included in the analysis. This number is well above the number of teams (the level of analysis) needed for the planned multilevel analyses (i.e., around 20 teams) [40]. The final number of teams and workshops conducted will be determined jointly by practice and academic partners based on the demand from the social service organizations, the number of work teams participating in each workshop, and the variation in types of EBIs that they plan to implement.

#### Workshops

During the workshops, the participating work teams will be offered hands-on support in using the structured decision support, and data for the research will also be collected (see below). Each work team will be offered the chance to participate in one to three workshops; the exact number will be determined after piloting the decision support. With guidance from the structured decision support, the work teams will initially identify benefits and risks of the EBI they intend to implement. They will also identify how relevant outcomes of the EBI can be measured. In the next step, the work team will identify the core components and outline the program logic of the EBI. Moreover, they will work with identifying key contextual characteristics of both the context in which the EBI was previously used and the context in which it will be used, along with identifying previously used implementation strategies for the EBI. Once the EBI, context, and implementation strategies have been explicated, the focus will be on deciding what needs to be adhered to and determining what can be adapted in the EBI. This step can help to identify adaptations needed for the EBI to work in a specific setting. This will result in a prototype that can be tested in practice and further adapted if needed. The decision support emphasizes careful documentation and evaluation of the testing, which will also be emphasized in the workshops.

The pedagogy of the workshops will be based on the theory of experiential learning [41]. The goal is to offer a good learning environment to increase the transfer of learning from the workshops to the workplace. This implies that concrete experiences are reflected upon, which will advance the understanding of theoretical concepts relevant to personal experience. Furthermore, an advanced understanding of the theoretical concepts will be translated into new actions, leading to new personal experiences [41].

One pedagogical aspect is also peer learning across participating units because several work teams (max. 10) will participate in one series of workshops. Because the groups can choose which EBI they will focus on, the groups might be working with different EBIs, which will further increase learning opportunities. Between the workshops, they will have assignments to anchor the planned work with their colleagues (i.e., inform the others at the unit or conduct small, practical testing).

#### Data collection

We use three main sources of data in connection with the workshop series: focus group interviews, questionnaire surveys, and participants’ documentation from workshops. These will be repeated at several occasions in conjunction with the workshop series. The workshops have a dual focus: to facilitate the management of adherence and adaptations and to provide a data source for answering the research questions. The golden rule for the data collection will be that it should provide value to the participants and researchers while placing as little of a burden on respondents as possible. We will follow Richter et al.’s methodology to evaluate the change process during the workshops with both qualitative and quantitative data [42]. The collection of qualitative data will be carried out in accordance with the COREQ checklist [43] (Additional file 1).

### Focus groups

Focus group interviews will be conducted at the start and end of each workshop series. The focus group format enables multiple perspectives and is believed to spark beneficial discussions on this complex and rather abstract topic [44]. All of the workshop participants will be invited to the focus groups; consequently, different types of social service organizations and professionals will be represented. The data from the first focus groups will be used to answer RQ1 concerning how adherence and adaptations are currently managed and how the participants perceive the cognitive and emotional demands of the dilemma. The combined data from the first and last focus group interviews will be used to answer the research questions of a longitudinal nature (i.e., whether the decision support decreases the demands [RQ2e] and whether the attitudes toward research changed during participation in the project [RQ3]). During the last focus group interview, the participants will also be asked to reflect on the data generated during the first focus group and during the workshop series using a sequential explanatory mixed-method approach [45]. This approach can enrich the interpretation and understanding of the different data sources and provide reflection opportunities for the participants. The focus group interviews will be based on the methodology proposed by Kitzinger [44]. For instance, the preferred number of participants will be four to eight persons, and the length will be approximately 60–120 min.

### Questionnaires

A questionnaire will be distributed to all participants at each workshop. It will be used to answer several of the research questions: The impact of the decision support (RQ2a–e) will be evaluated through the questionnaires. Furthermore, the questionnaires, together with the focus groups, will be used to answer RQ3 regarding whether participation in the project affected the participants’ attitudes toward research. In line with our previous procedure, some questions will be the same across all time points (e.g., the experience of combining different knowledge sources), whereas others will be specific to certain time points. This is to ensure that we capture the process of change at the most valid time point, without burdening the participants with lengthy questionnaires.

The research team will start by identifying suitable constructs to be measured, which will be discussed with representatives from the social service organizations. Thereafter, the research team will select the scales to be used in the questionnaire and, if needed, develop new items. Previously validated scales will be used as much as possible and could include the Copenhagen Psychosocial Questionnaire (COPSOQ), Intervention Process Measure (IPM), and Evidence-Based Practice Attitude Scale (EBPA).

### Documentation

Documentation produced by the participants during the workshops will be used to answer the research questions related to the impact of the decision support (RQ2a–c). Through the participants’ documentation on the worksheets, we will be able to collect data on how the participants identified core components in the EBIs applied, contextual differences, the types of adaptations made, and the extent to which they align with the goals of the EBIs. We will photograph the notes made by the teams at the end of each workshop.

#### Analyses

The data will be continuously analyzed for each specific RQ. Two members of the research team (HH and HG) will be responsible for the data analysis. The remaining research group members will act as informed outsiders. They will participate in iterative debriefing sessions to support the analysis and interpretation of the findings. For the focus group and the documentation data, the data will be analyzed with thematic analyses following the steps outlined by Braun and Clarke [46]. The focus groups will be audiotaped and transcribed verbatim. NVivo will be used for the data analysis.

The quantitative data will be analyzed through descriptive analyses (e.g., frequencies, correlations) and more complex analyses, such as multilevel modeling. This is used to account for the dependence of the data that is created by employees being nested within work units. SPSS 23, HLM 7.1, and Mplus 7.2 will be used. To have enough statistical power to conduct the multilevel analyses, we will follow the recommendation of Hox et al. [40] that 20 higher-order units—e.g., work units—will be sufficient to analyze the data.

## Discussion

The current study aims at investigating the adherence and adaption dilemma in social service and exploring how a structured decision support impacts how the dilemma is managed. Thus, our ambition is to contribute with novel knowledge on the adherence and adaption dilemma in general, as well as on the management of this dilemma in social service in particular. As such, the study is a contribution to implementation science, social service research, and social service practice.

An increased understanding of the balance between adherence and adaptation when using EBIs can be essential for new EBIs that are being developed and expected to be put to use in practice. Previous research indicates that adaptions of EBIs are common and that the dilemma tends to be managed in a non-reflective manner. In essence, improved knowledge about adherence and adaptations will increase the likelihood that service users will receive services that are in line with the best available evidence. The knowledge gained from the study is valuable beyond the specific field of social service practice. Insights made from the study might thus be useable in other contexts in which EBIs are implemented and the balance between adaption and adherence is managed by professionals.

The structured decision support that will be developed and tested has the potential to guide professionals in managing the balance between adherence to and adaptations of EBIs. Current research offers limited practical guidance for professionals’ management of the dilemma, and calls for practical tools directed toward professionals have been made [29]. New knowledge will be obtained regarding whether this type of decision support can improve professionals’ opportunities to identify the core components in an EBI and differences between their own context and the context in which the EBI has been used, as well as to reflect upon the necessary implementation strategies. In addition, we will test if the decision support can teach the professionals to reflect upon the opportunity to obtain the intended (and unintended) impact of an EBI depending on the balance of adherence and adaptations. These aspects are central prerequisites for making conscious choices regarding adherence and adaptations and conducting adaptations that align with the EBI’s goals. Thus, the project has the opportunity to produce a tool that can contribute to more careful adaptations, which in the longer run will improve the outcomes for service users. This will also be a contribution to the research, because this intervention can be further tested in future research.

In addition, the project contributes to the knowledge of how adherence and adaptation relate to social service professionals’ experience of cognitive and emotional demands at work. Currently, the dilemma is left to the professionals to solve, but without support, this has shown to be difficult to do without negative effects for professionals and service users alike: for professionals, for whom the dilemma has become a cognitive and emotional challenge that they have to face without sufficient resources, and for service users, who risk receiving less efficient, less safe, and less equal services. Therefore, the adherence and adaptation dilemma might have implications not only for the users of social services but also for the working conditions of social service professionals.

Furthermore, the project has the potential to help professionals with the fundamental principles of evidence-based practice (e.g., the use of research evidence together with the professionals’ own expertise and the service users’ needs). This is one of the burning challenges for staff in social services, yet little guidance is currently available. The project’s contributions lie in offering hands-on guidance for the professionals so that the EBIs (i.e., external research knowledge) can work better in their local context.

The project might also contribute to how social service professionals view research and scientific knowledge. It is possible that the collaborative research design, which starts from the needs and resources of the social service organizations and will be designed through interactions with the local stakeholders as the project unfolds, will impact how these stakeholders perceive research and their own opportunities to impact research conducted in social services. This would be an important contribution for advancing social service professionals’ orientation toward evidence-based practice and their own opportunities to impact the research conducted in the field.

## Supplementary information

**Additional file 1.** Consolidated criteria for reporting qualitative studies (COREQ).

## Data Availability

The datasets used will be available from the corresponding author on reasonable request.

## Abbreviations

EBI: Evidence-based intervention
R&D units: Research and development units
