# Supplementary material for: How can evidence-based interventions give the best value for users in social services? Balance between adherence and adaptations: a study protocol
Source: Implement Sci Commun. 2020 Feb 25;1:15. doi: 10.1186/s43058-020-00005-9 (PMC7427905; doi:10.1186/s43058-020-00005-9)
Supplement: Supplementary file 1 — Additional file 1. Consolidated criteria for reporting qualitative studies (COREQ). [file 43058_2020_5_MOESM2_ESM.docx]

**Additional file 1. Consolidated criteria for reporting qualitative studies (COREQ):**

**32-item checklist**

**Domain 1: Research team and reflexivity**

*Personal Characteristics*

1. Interviewer/facilitator: Which author/s conducted the interview or focus group?

2. Credentials: What were the researcher’s credentials? E.g. PhD, MD

3. Occupation: What was their occupation at the time of the study?

4. Gender: Was the researcher male or female?

5. Experience and training: What experience or training did the researcher have?

*Relationship with participants*

6. Relationship established: Was a relationship established prior to study commencement?

7. Participant knowledge of the interviewer: What did the participants know about the researcher? e.g. personal goals, reasons for doing the research

8. Interviewer characteristics: What characteristics were reported about the interviewer/facilitator? e.g. Bias, assumptions, reasons and interests in the research topic

**Domain 2: study design**

*Theoretical framework*

9. Methodological orientation and Theory: What methodological orientation was stated to underpin the study? e.g. grounded theory, discourse analysis, ethnography, phenomenology, content analysis

*Participant selection*

10. Sampling: How were participants selected? e.g. purposive, convenience, consecutive, snowball

11. Method of approach: How were participants approached? e.g. face-to-face, telephone, mail, email

12. Sample size: How many participants were in the study?

13. Non-participation: How many people refused to participate or dropped out? Reasons?

*Setting*

14. Setting of data collection: Where was the data collected? e.g. home, clinic, workplace

15. Presence of non-participants: Was anyone else present besides the participants and researchers?

16. Description of sample: What are the important characteristics of the sample? e.g. demographic data, date

*Data collection*

17. Interview guide: Were questions, prompts, guides provided by the authors? Was it pilot tested?

18. Repeat interviews: Were repeat interviews carried out? If yes, how many?

19. Audio/visual recording: Did the research use audio or visual recording to collect the data?

20. Field notes: Were field notes made during and/or after the interview or focus group?

21. Duration: What was the duration of the interviews or focus group?

22. Data saturation: Was data saturation discussed?

23. Transcripts returned: Were transcripts returned to participants for comment and/or correction?

**Domain 3: analysis and findings**

*Data analysis*

24. Number of data coders: How many data coders coded the data?

25. Description of the coding tree: Did authors provide a description of the coding tree?

26. Derivation of themes: Were themes identified in advance or derived from the data?

27. Software: What software, if applicable, was used to manage the data?

28. Participant checking: Did participants provide feedback on the findings?

*Reporting*

29. Quotations presented: Were participant quotations presented to illustrate the themes / findings? Was each quotation identified? e.g. participant number

30. Data and findings consistent: Was there consistency between the data presented and the findings?

31. Clarity of major themes: Were major themes clearly presented in the findings?

32. Clarity of minor themes: Is there a description of diverse cases or discussion of minor themes?
